# *Neohexostoma gymnosardae* n. sp. (Monogenea, Hexostomatidae), a gill parasite of *Gymnosarda unicolor* (Valenciennes) (Teleostei, Scombridae) in the South China Sea

**DOI:** 10.1051/parasite/2020067

**Published:** 2020-12-11

**Authors:** Pei-Wei Zhu, You-Zhi Li, Lin Liu, Xue-Juan Ding, Kai Yuan

**Affiliations:** 1 Guangzhou Key Laboratory of Subtropical Biodiversity and Biomonitoring, Guangdong Provincial Key Laboratory for Healthy and Safe Aquaculture, College of Life Science, South China Normal University Guangzhou 510631 China

**Keywords:** Monogenea, Hexostomatidae, New species, *Gymnosarda unicolor*, the South China Sea

## Abstract

Parasite biodiversity of fish in coral reefs of the South China Sea is still incompletely explored. We describe here a new species of *Neohexostoma* (Monogenea: Hexostomatidae) from the gill filaments of the dogtooth tuna *Gymnosarda unicolor* (Scombridae), collected off Yongshu Reef, South China Sea. *Neohexostoma gymnosardae* n. sp. is distinguished from its congeners by the following features: (i) haptor clearly marked from body proper by a strongly constricted peduncle, divided in its posterior margin into two symmetrical lobes, (ii) vagina armed with scattered small blunt spines, (iii) eggs tied by their long polar filaments, (vi) esophagus with several lateral diverticula, (v) intestinal ceca unfused and extending into the haptor. We present an analysis of the relationships of this monogenean based on partial 28S rDNA sequences. An identification key for species of *Neohexostoma* is provided. This is the first member of the genus *Neohexostoma* known to parasitize a species of *Gymnosarda*.

## Abstract (fr)

La biodiversité parasitaire des poissons dans les récifs coralliens de la mer de Chine méridionale est encore incomplètement explorée. Nous décrivons ici une nouvelle espèce de *Neohexostoma* (Monogenea, Hexostomatidae) des filaments branchiaux du thon à dents de chien *Gymnosarda unicolor* (Scombridae), collecté au large du récif de Yongshu, mer de Chine méridionale. *Neohexostoma gymnosardae* n. sp. se distingue de ses congénères par les caractéristiques suivantes : (i) hapteur clairement séparé du corps proprement dit par un pédoncule fortement resserré, divisé dans sa marge postérieure en deux lobes symétriques, (ii) vagin armé de petites épines émoussées éparses, (iii) œufs attachés par leurs longs filaments polaires, (vi) œsophage avec plusieurs diverticules latéraux, (v) caeca intestinaux non fusionnés et s’étendant dans le hapteur. Nous présentons une analyse des relations de ce monogène basée sur des séquences partielles d’ADNr 28S. Une clé d’identification des espèces de *Neohexostoma* est fournie. Ceci est le premier membre du genre *Neohexostoma* connu pour parasiter une espèce de *Gymnosarda*.

## Introduction

*Gymnosarda unicolor* Rüppell 1836 (Perciformes: Scombridae) is an epipelagic, coral-reef associated fish endemic to the Indo-Pacific region [2]. It is the sole species of the genus *Gymnosarda* [2]. Very little is known about the biology and ecology of *G. unicolor*. It is almost exclusively solitary, associated with reef structures, and is rarely found in the open sea or in schools [6]. Coral reefs are known for their very high level of biodiversity [13]. However, parasite biodiversity in the coral reefs of the South China Sea is still incompletely explored.

The Hexostomatidae Price, 1936 (Monogenea: Mazocraeidea) includes 18 species in 4 genera: *Hexostoma* Rafinesque, 1815; *Homostoma* Unnithan, 1965; *Neohexostoma* Price, 1961; and *Unnithania* Gupta & Sachdeva, 1988, all parasites of marine fishes, mainly Scombridae [21, 26, 30, 31]. Boeger and Kritsky [3] conducted a phylogenetic analysis of the Monogenea using morphological characters and proposed the new suborder Hexostomatinea within the Mazocraeidea to accommodate Hexostomatidae. In the South China Sea, two valid species of Hexostomatidae have been reported: *H. auxisi* Palombi, 1943 from *Auxis thazard* and *N. euthynni* (Meserve, 1938) Price, 1961 from *Euthynnus affinis* and *Auxis thazard* [36]. In the course of a parasitological study of monogeneans of marine fishes off the South China Sea, we collected representatives of an undescribed species of *Neohexostoma* on the gills of *Gymnosarda unicolor*. The species is described here. An identification key to *Neohexostoma* is also provided.

## Materials and methods

### Fishes

Throughout May 2017, three *G. unicolor* were collected at Yongshu Reef (9°33′N, 113°E) off the South China Sea. Fish specimens were transferred to the laboratory and identified using keys [11]. Fish specimens were photographed prior to removing gills. Gills were removed from each fish and observed under the microscope for the presence of monogeneans.

### Morphological methods

Gills were excised and placed in Petri dishes with sea water and examined for monogeneans with the aid of a stereomicroscope. Monogeneans were removed alive from gills using fine dissection needles. Four specimens were preserved in 70% ethanol, stained with acetic carmine, dehydrated in graded ethanol series (70%, 96% and 100%), cleared in clove oil, and mounted in Canada balsam. Two specimens were directly mounted in Berlese’s fluid to study the morphology of clamps and the genital atrium. Illustrations were drawn with the help of the drawing apparatus of an Olympus BX51 microscope (Olympus Corporation, Japan), and then redrawn on a computer with Photoshop CS4.0 (Adobe, USA). Measurements were made using Olympus DP22 software. Measurements are in micrometres (μm), and indicated as the range followed by the mean and the number of measurements in parentheses.

### Molecular methods

A single specimen was fixed in absolute ethanol then subjected to molecular analyses. Total genomic DNA was extracted using a TIANamp Marine Animal DNA Kit (Tiangen Biotech, China), following the manufacturer’s instructions. The partial C1-D2 domain of 28S rDNA was amplified with PCR using previously published primer pairs (C1F: 5′–ACCCGCTGAATTTAAGCAT–3′ and reverse primer D2R: 5′–TGGTCCGTGTTTCAAGAC–3′) [10]. Each PCR amplification was performed in a 50-μL volume containing 5 μL of DNA template, 25 μL of Master Mix (dNTP, 2× buffer, Taq polymerase), 2 μL of each primer, and 16 μL of double-distilled water, under the following conditions: initial denaturation at 95 °C for 5 min; followed by 35 cycles of 95 °C for 30 s, 55 °C for 30 s, 72 °C for 1 min; and a final elongation at 72 °C for 10 min. PCR products were detected by 1% agarose gel electrophoresis and sequenced by the Sangon Biotech Company (Shanghai, China). The sequences obtained were analyzed using DNAMAN 7.0 software (Lynnon Biosoft, USA), compared to GenBank database content with BLAST, and deposited in GenBank under the accession number MN242399.

### Trees and distances

Sequences of 10 species belonging to 6 families available in GenBank, and one sequence of *Neohexostoma gymnosardae* n. sp. generated in this study were included in the phylogenetic analyses (Table 1). Evolutionary analyses were conducted in MEGA 7.0. [18]. The trees were inferred using the maximum likelihood (ML) method and the neighbor-joining method, with a sequence of *Polystoma gallieni* Price, 1938 as the outgroup [8, 28]. For the ML tree, the best model, estimated by MEGA7, was the general time-reversible model with discrete gamma distribution (GTR + G). The tree was re-sampled with 1000 bootstrap replicates to evaluate the reliability of the groups. Kimura 2-parameter distances between sequences were estimated with MEGA7.0 [16].

**Table 1:** Species of monogeneans used in the molecular analyses.

Species	Family	Accession No.	Reference
*Neohexostoma gymnosardae* n. sp.	Hexostomatidae	MN242399	Present study
*Hexostoma thynni* (Delaroche, 1811) Rafinesque, 1815	Hexostomatidae	EF653383	[1]
*Diplostamenides sciaenae* (Goto, 1894) Mamaev, 1986	Microcotylidae	FJ432589	Direct submission
*“Cynoscionicola branquialis”*	Microcotylidae	AF382050	[23]
*Diclidophora denticulata* (Olsson, 1876) Price, 1943	Diclidophoridae	AF382047	[23]
*Urocotyle nibae* Zhang & Xiao in Zhang, Yang & Liu, 2001	Diclidophoridae	FJ432588	Direct Submission
*Gotocotyla bivaginalis* (Ramalingam, 1961) Rohde, 1976	Gotocotylidae	AF382039	[23]
*Gotocotyla secunda* (Tripathi, 1954)	Gotocotylidae	AF382040	[23]
*Pseudohexabothrium taeniurae* Agrawal, Chisholm & Whittington, 1996	Hexabothriidae	AF382035	[23]
*Hypanocotyle bullardi* Chero, Cruces, Sáez, Camargo, Santos & Luque, 2018	Hexabothriidae	MG591249	[5]
*Polystoma gallieni* Price, 1938	Polystomatidae	AF382064	Direct Submission

“*Cynoscionicola branquialis*” was accepted as “*Cynoscionicola branchialis*”, but in a status of taxon inquirendum. *Gotocotyla secunda* (Tripathi, 1954) was accepted as *Gotocotyla acanthura* (Parona & Perugia, 1896) Meserve, 1938.

## Results

### Molecular analyses

**Figure 1:**
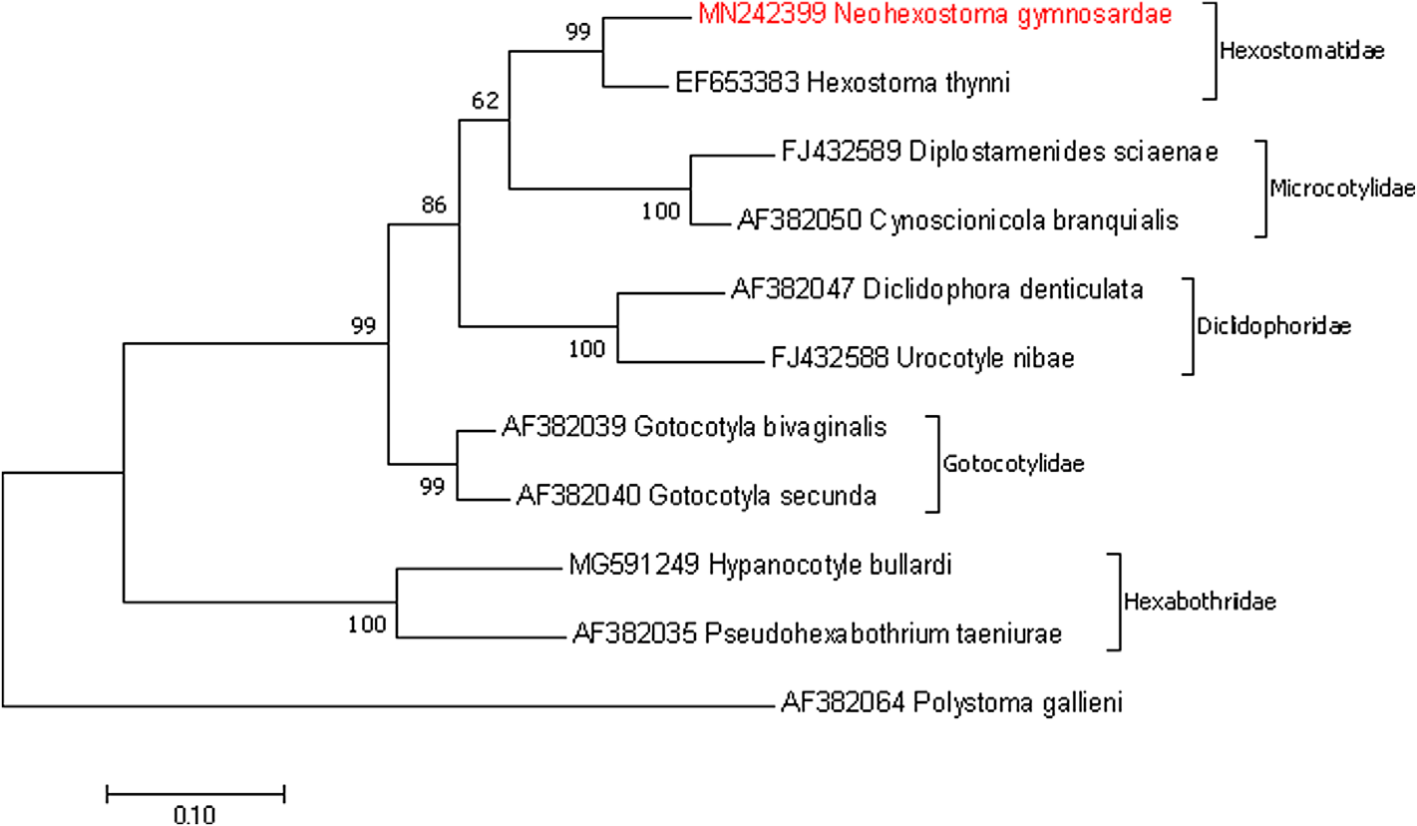
Maximum likelihood tree based on an analysis of 28S rDNA sequences. Bootstrap percentages with 1000 replicates.

28S rDNA sequence data with 881 bp for *N. gymnosardae* n. sp. was generated, and the BLAST result indicated a 92.42% identity with 98% coverage for *H. thynni* (Delaroche, 1811) Rafinesque, 1815 (EF653383), and less than 85% identity with 100% coverage for other monogeneans in GenBank. For trees and genetic distances, there was a total of 837 positions in the final dataset, including 388 conserved sites, 449 variable sites, and 301 parsimony informative sites. The species most closely related to *N. gymnosardae* n. sp. was *H. thynni*, with Kimura two-parameter distance of 8.2% (Table 2). The neighbour-joining and maximum likelihood methods led to identical tree topologies and thus only the ML tree is shown (Fig. 1). The tree showed *N. gymnosardae* n. sp. grouping with *H. thynni* with a statistical support of 100% in the clade Hexostomatidae.

**Table 2:** Estimates of evolutionary divergence between sequences (Kimura-2 parameter model), shown as percentages.

		1	2	3	4	5	6	7	8	9	10	11
1	MN242399*N. gymnosardae*											
2	EF653383*H. thynni*	8.2										
3	FJ432589*D. sciaenae*	23.0	21.1									
4	AF382050*C. branquialis*	22.3	20.9	6.9								
5	AF382039*G. bivaginalis*	18.6	18.9	27.4	25.8							
6	AF382040*G. secunda*	20.4	20.2	26.8	25.5	5.0						
7	AF382047*D. denticulata*	24.6	22.8	25.1	22.3	23.0	22.3					
8	FJ432588*U. nibae*	25.6	22.6	27.6	25.1	23.2	22.8	13.6				
9	MG591249*H. bullardi*	43.0	41.9	46.3	44.3	37.5	37.1	41.4	46.7			
10	AF382035*P. taeniurae*	42.7	42.5	47.2	44.9	37.4	39.6	45.6	46.7	19.0		
11	AF382064*P. gallieni*	62.3	57.2	65.1	62.2	51.5	51.8	62.5	59.4	60.9	56.9	

Family Hexostomatidae Price, 1936

Genus *Neohexostoma* Price, 1961

#### *Neohexostoma gymnosardae* n. sp.

urn:lsid:zoobank.org:act:E20B680B-EA84-4F93-96CC-1D2393A79C51

Type-host: *Gymnosarda unicolor* Rüppell, 1836 (Perciformes: Scombridae, dogtooth tuna).

Site of infection: Gills.

Type-locality: Yongshu Reef (9°33’N, 113°E), South China Sea, Western Pacific Ocean.

Prevalence: 3 of 3 hosts infected (100%) with a total of 10 worms.

Type material: Holotype (LFP. 2017050801), four paratypes (LFP. 2017050802–05), Laboratory of Fish Parasite, College of Life Science, South China Normal University, Guangzhou, China. One paratype (NHMUK No. 2019.10.30.3), Natural History Museum, London (NHMUK).

Etymology: The species is named after its host.

##### Description (Figs. 2 and 3)

**Figure 2:**
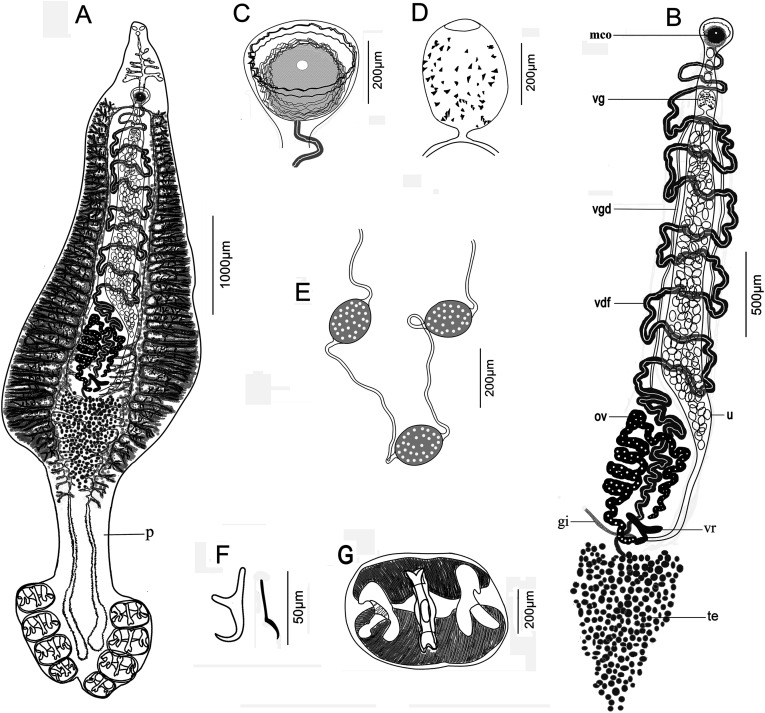
*Neohexostoma gymnosardae* n. sp. from gills of *Gymnosarda unicolor*. (A) Whole worm, ventral view (p, peduncle); (B) Reproductive system (MCO, male copulatory organ; vg, vagina; vgd, vaginal ducts; vdf, vas deferens; ov, ovary; vr, vitelline reservoir; gi, genitointestinal canal; u, uterus; te, testes); (C) Male copulatory organ; (D) Vagina; (E) Eggs; (F) Anchors; (G) Clamp with sclerites in front view.

**Figure 3:**
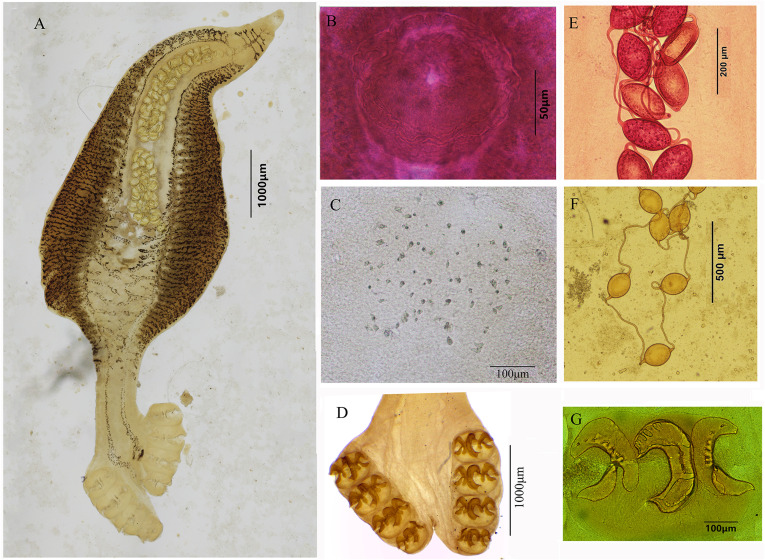
Photographs of *Neohexostoma gymnosardae* n. sp. (A) Holotype, whole worm (ventral view); (B) Male copulatory organ; (C) Vaginal spines; (D) Haptor; (E)–(F) Eggs; (G) Clamp with sclerites in lateral view. (B)–(G) are paratypes.

Based on six whole-mounted worms. Body elongate, divided into anterior body proper, peduncle and haptor; body proper tapering anteriorly, followed by a strongly constricted peduncle, and then a broadening haptor (Figs. 2A, 3A). Body total length 9660–18,800 (13,827; *n* = 6), greatest width 2875–5375 (3847; *n* = 6) at level of ovary. Peduncle 929–1690 (1324; *n* = 5) long, 566–1021 (824, *n* = 5) wide, from the termination of testes to the anterior of haptor. Haptor somewhat irregular, clearly marked from body proper by a peduncle and divided by a split in its posterior margin (Fig. 3D), 1172–1810 (1446, *n* = 6) long from the first pair of clamps to the fourth pair, 1369–2582 (1827, *n* = 6) wide at level of first pair of clamps. Haptor with four pairs of clamps arranged in two longitudinal rows, most anterior pair 505–697 (594, *n* = 6) long, 275–506 (415, *n* = 6) wide, second pair 577–713 (620, *n* = 6) long, 327–477 (395, *n* = 6) wide, third pair 475–624 (587, *n* = 6) long, 302–455 (376, *n* = 6) wide, and fourth pair 389–496 (447, *n* = 6) long, 233–358 (316, *n* = 6) wide. Each clamp consisting of one middle sclerite and two pairs of lateral sclerites longitudinally imbedded in two muscular pads; middle sclerite X-shaped in front view (Fig. 2G) or C-shaped in lateral view (Fig. 3G); two lateral sclerites inverted Y-shaped. Haptor with two pairs of anchors (Fig. 2F), large anchors 43–57 (50, *n* = 4) long, with a long root and well curved blade; inner anchors sickle shaped, 39–42 (41, *n* = 2) long. One pair of prohaptoral suckers elliptical, 62–99 (83, *n* = 5) long, 51–102 (70, *n* = 5) wide. Pharynx small, 63–93 (82, *n* = 3) long, 49–58 (55, *n* = 3) wide. Esophagus 700–2691 (1352, *n* = 5) long, with several lateral branches, bifurcating immediately posterior to genital atrium into two intestinal ceca (Fisg. 2A, 3A). Intestinal ceca extending into the haptor, not united posteriorly, with numerous digitiform lateral diverticula and several medial diverticula in ovarian and testicular region, but without branches in peduncle and haptor region (Figs. 2A, 3A). Testes rounded, numerous, 185–246 (218, *n* = 4), packed together in posterior part of ovary, not extending into peduncle (Fig. 2B). Vas deferens arising from testes and passing medially from ovary, then winding strongly forward dorsal to uterus (Figs. 2A, 2B). Genital atrium forming a globular sucker, with muscular wall enwrapping male copulatory organ (MCO), at a distance 706–2510 (1699, *n* = 5) from head end. MCO, cup-shaped with a bulbous muscular cirrus covered with ring-like corrugated edge (Figs. 2C, 3B), 92–321 (225, *n* = 5) long, 112–348 (228, *n* = 5) wide. Ovary sinuous, roughly inverted U-shape, with limbs greatly convoluted (Fig. 2B), measuring 1150–1650 (1444, *n* = 4) long. Vagina opening mediodorsally, behind genital pore, 244–359 (301.5, *n* = 2) long, 191–250 (220.5, *n* = 2) wide, armed with sparsely small spines (Figs. 2D, 3C). Two parallel vaginal ducts, conspicuous, running back along sides of uterus. Vitellaria follicles distributed along intestinal ceca. Vitelline reservoir Y-shape. Uterus arising along left side of ovary, then midventral distended with numerous eggs (Fig. 2B). Genitointestinal canal enters right intestinal cecum. Oviduct and oötype not observed; precise junctions between vitelline reservoir, ovary, uterus and genitointestinal canal not elucidated. Eggs oval, 125–193 (156, *n* = 5) long, 91–137 (114, *n* = 5) wide, joined in a chain by their filaments, filaments 411–719 (518, *n* = 3) long (Figs. 2E, 3E, 3F).

##### Differential diagnosis

Monogeneans found on the gills of *G. unicolor* are allocated to *Neohexostoma* by the following morphological features: body elongate, widest in ovarian region; haptor with four pairs of clamps arranged as two more or less vertical rows, decreasing in size anteroposteriorly; vitellarium not extending posteriorly beyond the distal portion of the testes [26]. *Neohexostoma* as presently constituted includes seven species: *N. thunninae* (Parona & Perugia, 1889) Price, 1961, *N. euthynni* (Meserve, 1938) Price, 1961, *N. extensicaudum* (Dawes, 1940) Price, 1961, *N. pricei* (Koratha, 1955) Price, 1961, *N. robustum* Price, 1961, *N. kawakawa* Yamaguti, 1968, and *N. mochimae* Fuentes-Zambrano, 1997 [32]. The new species can be distinguished from all *Neohexostoma* spp. by the shape of the haptor, the armature of the vagina, and the assembly of eggs. The haptor is divided into symmetrical lobes by a slender split in its posterior margin, the vagina is armed with scattered spines, and the eggs are tied by their long polar filaments.

Apart from the above, the new species can also be distinguished from its congeners by its body shape, intestine, and clamp disposition. *Neohexostoma gymnosardae* n. sp. is characterized by a well visible peduncle: of the seven species of *Neohexostoma* mentioned above, only *N. thunninae* and *N. mochimae* have a constriction at the beginning of the haptor, separating the latter from the rest of the body [24, 25, 35]. The new species most closely resembles *N. thunninae* by its body shape and the possession of a peduncle. *Neohexostoma gymnosardae* n. sp. differs from *N. thunninae* by having an esophagus with lateral diverticula, ceca extending more posteriorly into the haptor and the post-atrial intestines bifurcating. Moreover, in *N. gymnosardae* n. sp. the anchors are placed between clamps of the most posterior pair, whereas they are borne on a short lappet in *N. thunninae* [24, 25].

*Neohexostoma gymnosardae* n. sp can be distinguished from *N. kawakawa*, *N. mochimae* and *N. euthynni* by having testes tightly packed into a post-ovarian pile [20, 34, 35]. Furthermore, in *N. gymnosardae* n. sp., intestinal bifurcation is post-atrial and the unfused intestinal ceca extend into the haptor; in *N. kawakawa* and *N. euthynni*, the intestinal bifurcation is pre-atrial and ceca are united posteriorly [20, 34]. *Neohexostoma gymnosardae* n. sp. differs from *N. pricei* by the extension of intestinal ceca into the haptor (up to a shorter distance in *N. pricei*) and the shape of lateral sclerites of clamps (straight in *N. pricei* vs. double-pronging in *N. gymnosardae* n. sp.) [17]. *Neohexostoma gymnosardae* n. sp. can be further distinguished from *N. extensicaudum* and *N. robustum* by body shape and clamp disposition. Both *N. extensicaudum* and *N. robustum*, like most of *Neohexostoma* spp., have an elongated waist-like constriction in the testicular region, which is absent in the new species [7, 26]. Moreover, clamps of the right row in *N. extensicaudum* lie very close to those of the left row so that the posterior end of the body is much more attenuated than is the case in other species [7]; and in *N. robustum*, clamps are arranged in oblique transverse rows on a roughly triangular haptor [26].

The comparison of morphometrics of *Neohexostoma gymnosardae* n. sp. and *Neohexostoma* spp. is presented in Table 3. *Neohexostoma gymnosardae* n. sp. most closely resembles its congeners by having clamps decreasing in size anteroposteriorly. It differs from *N. euthynni*, *N. kawakawa*, and *N. mochimae* by the number of testes, host and locality for the latest species. In addition, *N. gymnosardae* n. sp*.* can be distinguished from *N. kawakawa*, *N. euthynni* and *N. extensicaudum* by having a longer polar filament. We note that body length in *N. gymnosardae* n. sp*.* is greater than in *N. euthynni*, *N. pricei*, *N. mochimae*, and *N. kawakawa*. However, such a measurement is generally not a reliable characteristic for species differentiation [4]. We present herein a key to species of *Neohexostoma* as follows, which is modified from Millemann [21] and Zambrano [35].

**Table 3:** Measurements of *Neohexostoma gymnosardae* n. sp. from *Gymnosarda unicolor* from the South China Sea, and *Neohexostoma* spp.

	*N. gymnosardae* n. sp.	*N. mochimae* Fuentes-Zambrano, 1997	*N. kawakawa* Yamaguti, 1968	*N. thunninae* (Parona & Perugia, 1889)	*N. euthynni* (Meserve, 1938)	*N. extensicaudum* (Dawes, 1940)	*N. robustum* Price, 1961	*N. pricei* (Koratha, 1955)
Hosts	*Gymnosarda unicolor*	*Auxis thazard*	*Euthynnus yaito Neothunnuus macropterus*	*Thynnus thunninae*	*Euthynnus alleteratus* [*Euthynnus lineatus*]	*Thunnus thynnus*	*Thunnus obesus (Parathynnus sibi)*	*Sarda sarda*
Locality	South China Sea, P.	Venezuela, A.	Hawaii, P.	Italy, M.	Galapagos Islands, P. [Baja California, P.]	English Channel, A.	Tropical Pacific	Texas, A.
Source	Present study	[35]	[34]	[24, 25]	[20] [21]	[7]	[26]	[17]
Body length	9660–18800 (13827)	4444–6166 (5093)	4900–8400	11,000–12,000	5853 [3570–5850]	11,000	17,000	4500
Body width	2875–5375(3847)	874–1160 (1011)	70–180	2000	953 [740–950]	3300	4000–4700	400–850
Haptor length	1172–1810 (1 446)	665–1140 (903)			953			440
Haptor width	1369–2582 (1827)	1273–1615 (1444)	1200–1550	1500	1300			750–850
Clamps	1st pair: 275–506 × 505–697 (415 × 594)	Anterior 3 clamps: 122–209 × 200–315 (164 × 250)	224–370 × 200–230	1st pairs: 275 × 220	203 × 339 [Anterior 3 clamps: 153–255 × 221–403, posterior-most pair: 156–238 × 194–332]	0.067 **	1st pair: 500 × 750	Anterior two pairs: 500 × 340
	2nd pair: 327–477 × 577–713 (395 × 620)	Posterior clamp: 94–177 × 117–198 (126 × 166)		2nd pairs: 225 × 180		0.030***	2nd pair: 600 × 850	3rd pair: 460 × 340
	3rd pair: 302–455 × 475–624 (376 × 587)						3rd pair: 500 × 670	4th pair: 375 × 300
	4th pair: 233–358 × 389–496 (316 × 447)						4th pair: 350 × 500	
Oral sucker	62–99 × 51–102 (83 × 70)	29–30 × 28–30(30 × 29)	28–45 *		56 × 40[27–56 × 24–40]	– × 100		40 × 30
Pharynx	63–93 × 49–58 (82 × 55)	40–13 × 24–29 (42 × 26)	40–58 × 23–35		[44–68 × 26–36]	100 × 70		75 × 45
Vagina	244–359 × 191–250 (302 × 221)	10–40 (35) *	Pads: 60–80 × 20–30	54*		600 × 350		Right pad: 55 × 24
								Left pad: 70 × 24
Genital atrium	92–321 × 112–348 (225 × 228)		40–70 *			600 × 300		
Large anchor	43–57 (50)	34–90(64)	85–105	135	68 [85–120]	75	100	145
Small anchor	39–42(41)	20–31(25)	20–40	45	34 [24–34]	15	40	
Eggs	125–193 × 91–137 (156 × 114)	182–196 × 74 (196 × 74)	180–260 × 70–160	270 × 91	168–203 × 72–80 [103–221 × 44–105]	250 × 150	220 × 110	
Egg filaments	411–719 (518)		up to 200		100 [anterior: 100–179, posterior: 100–161]	250		
Testes number	185–246	30–35	13–35		26 [32–40]		Numerous	

The width of the body given for *N. extensicaudum* is that of the third region. The data for *N. euthynni* in square brackets are from Millemann (1956) [21].

* Diameter.

** Ratio large clamp/body length.

*** Ratio small clamp/body length.

A., Atlantic Ocean. M., Mediterranean Sea. P., Pacific Ocean.

##### Key to species of *Neohexostoma* (modified from Millemann [21] and Zambrano [35]):

1. Non-existent separation between the termination of testes and the anterior of haptor......………….2

Remarkable separation between the termination of testes and the anterior of haptor……………….3

2. Clamps similar in size…………………….…….……….………………………….… *N. kawakawa*

Clamps decreasing backwards in size…………………………………………………*N. mochimae*

3. A strong constriction before haptor………………………………………………………………4

No constriction before haptor ……………………………………………………………………….5

4. Haptor with a split in its posterior margin….………………………………. *N. gymnosardae*

Haptor with a short lappet…………………………………………………………*N. thunninae*

5. Most posterior pair of clamps approximately one-half the size of anterior pairs ……………………………………………………………………………………………….…*N. extensicaudum*

Most posterior pair of clamps slightly smaller than anterior three pairs ………………………….6

6. Eggs with two short polar filaments……………………………………………………. *N. robustum*

Eggs with two long polar filaments……………….……………………………………………….7

7. Lateral sclerites of clamps straight…………………………………………………………. *N. pricei*

Lateral sclerites of clamps double-pronged ……………………………………...………*N. euthynni*

## Discussion

This study describes a new species of *Neohexostoma, N. gymnosardae* n. sp., obtained from *Gymnosarda unicolor*, caught off Yongshu Reef, South China Sea, Northern Pacific. We note that we previously named this species *Leptohexostoma gymnosarda* n. gen. n. sp. in an abstract of a symposium held in China [19]. According to Article 9 for “What does not constitute published work” in the International Code of Zoological Nomenclature (ICZN), abstracts of articles, papers, posters, texts of lectures, and similar material when issued primarily to participants at meetings, symposia, colloquia or congresses does not constitute published work within the meaning of the Code [12]. The name, *Leptohexostoma gymnosarda*, proposed in the abstract is a *nomen nudum*.

Millemann synonymized *H. macracanthum* with *H. euthynni* and noted that all species of *Hexostoma* could be separated into two different morphological groups [9, 21]. In species assigned to the first group, clamps are arranged in straight transverse rows. Clamps of representatives of the remaining species are disposed in two longitudinal rows. Price [26] established *Neohexostoma* to accommodate three species transferred from *Hexostoma*, along with the newly described *N. robustum*. He also provided the main differences between the new genus and *Hexostoma.* Subsequently, Yamaguti [33] and Unnithan [30] amended the diagnoses of Hexostomatidae and that of *Neohexostoma*. All the previously mentioned studies valued the haptor and body shape as a generic feature. Our new species shares the “classic” morphological characteristics with species of *Neohexostoma* as stated in the differential diagnosis [29, 33].

The waist-like constriction in the testicular region, characteristic of *Neohexostoma* [26, 33], is not observed in the new species. Actually, this waist-like constriction was not mentioned in the original description of *N. thunninae* [24, 25]. Some species in *Hexostoma*, such as *Hexostoma dissimile* (Yamaguti, 1937) Sproston, 1946, *Hexostoma sibi* Yamaguti, 1968 and *Hexostoma grossum* (Goto, 1894) Sproston, 1946, also have a slightly constricted waist in the testicular region or in front of the haptor [22, 29, 33, 34]. Hence, we suggest that the soft parts of monogeneans should be treated with caution when defining a new taxon, as these features may be associated with the *in situ* position of the worm on the host. Although measurements of some body parts are conventionally used for the systematics of Monogenea, some measurements (e.g. size of body, size of clamps) vary widely within this new species and overlap with other species (Table 3), making determination to species level difficult. The sclerotized structures such as clamp sclerites are considered important taxonomical features for species identification. Generally, the middle skeletal piece in *Neohexostoma* spp. is X-shaped with a base plate [2]. However, clamp sclerites are actually complicated three-dimensional structures, resulting in different shapes observed from different sides (Fig. 2G vs. Fig. 3G).

Also noteworthy is the genital system. Based on previous descriptions and figures, although some authors failed to determine the number of testes in their descriptions, the number of testes in *Neohexostoma* is usually less than 50 and testes are arranged in two or three alternating rows [9, 27, 34, 35]. Abundant testes (185–246 in our new species) are usually seen in some species of *Hexostoma*, such as *H. grossum*, *H. sibi*, and *H. dissimile* [14, 29, 33, 34]. Moreover, the terminal portion of the vagina in members of the Hexostomatidae is typically represented by two symmetrical pads with densely conical spines forming two serrated edges, which is dissimilar to that in our species. Another point of interest is that the eggs of new species are joined in a chain by their long filaments. Monogeneans shows a remarkable diversity in the shape and size of eggs, as well as their appendages. Although this type of egg assembly is also observed in other monogeneans (e.g. *Squalonchocotyle catenulate* Guberlet, 1933) [15], it is unique in Hexostomatidae. Unfortunately, as a single sequence of *H. thynni* is available, we cannot further comment on the phylogenetic relationships within *Neohexostoma*.

## Conflict of interest

All authors have no conflict of interest. We acted in accordance with all applicable institutional and national laws and guidelines during this research.
